# Protective Effect of Liposome-Encapsulated Glutathione in a Human Epidermal Model Exposed to a Mustard Gas Analog

**DOI:** 10.1155/2011/109516

**Published:** 2011-05-30

**Authors:** Victor Paromov, Sudha Kumari, Marianne Brannon, Naga S. Kanaparthy, Hongsong Yang, Milton G. Smith, William L. Stone

**Affiliations:** ^1^Department of Pharmacology, Quillen College of Medicine, East Tennessee State University, Johnson City, TN 37614, USA; ^2^Department of Pediatrics, Quillen College of Medicine, East Tennessee State University, Johnson City, TN 37614, USA; ^3^Amaox, Ltd., 6300 N. Wickham Road 208, Melbourne, FL, USA

## Abstract

Sulfur mustard or mustard gas (HD) and its monofunctional analog, 2-chloroethyl ethyl sulfide (CEES), or “half-mustard gas,” are alkylating agents that induce DNA damage, oxidative stress, and inflammation. HD/CEES are rapidly absorbed in the skin causing extensive injury. We hypothesize that antioxidant liposomes that deliver both water-soluble and lipid-soluble antioxidants protect skin cells from immediate CEES-induced damage via attenuating oxidative stress. Liposomes containing water-soluble antioxidants and/or lipid-soluble antioxidants were evaluated using *in vitro* model systems. Initially, we found that liposomes containing encapsulated glutathione (GSH-liposomes) increased cell viability and attenuated production of reactive oxygen species (ROS) in HaCaT cells exposed to CEES. Next, GSH-liposomes were tested in a human epidermal model, EpiDerm. In the EpiDerm, GSH-liposomes administered simultaneously or 1 hour after CEES exposure (2.5 mM) increased cell viability, inhibited CEES-induced loss of ATP and attenuated changes in cellular morphology, but did not reduce caspase-3 activity. These findings paralleled the previously described *in vivo* protective effect of antioxidant liposomes in the rat lung and established the effectiveness of GSH-liposomes in a human epidermal model. This study provides a rationale for use of antioxidant liposomes against HD toxicity in the skin considering further verification in animal models exposed to HD.

## 1. Introduction

Sulfur mustard (military code HD) is a weapon of mass-destruction that is relatively simple to synthesize; it is lethal in high doses and causes severe damage to skin, lungs, respiratory tract, and eyes [1]. The most prominent toxic effects of HD are to dermal tissues where it produces severe damage including extremely slow healing lesions and blisters, which can ulcerate and promote secondary infections [2, 3]. Despite the long military history of HD, the molecular mechanisms for its toxicity are not fully elucidated and an effective countermeasure has remained elusive. Both HD and CEES (a monofunctional analog of sulfur mustard) are alkylating agents that covalently react with nucleic acids, proteins, and intracellular glutathione (GSH). GSH is a key intracellular antioxidant that is depleted by exposure to HD or CEES [4, 5]. A considerable body of evidence supports the notion that oxidative stress is an important factor in promoting HD/CEES toxicity and that antioxidants such as GSH or NAC could be important countermeasures [5–13]. 

Recent studies have shown that GSH and NAC, especially if used as a pretreatment, not only increase viability of CEES-treated keratinocytes, but also attenuate CEES toxicity in SKH-1 hairless mouse skin [14, 15]. The effectiveness of these antioxidants in cultured cells and animal models suggests that an oral application of GSH and/or NAC should be considered as a potential countermeasure to the HD/CEES toxicity in humans. However, certain difficulties of such approach should be taken to account. First, the antioxidants are effective only during a short period after the exposure (≤1 hour). Second, the effective dose of GSH in the animal experiments was 300 mg/kg [15], which suggests that the effective GSH dose in human should be in the range of few tens of grams. Therefore, a systemic application in humans would be difficult due to the requirement of large amounts of solid or liquid antioxidants for a single treatment. A topical application seems to be more appropriate; however, the absorption of water-soluble antioxidants is minimal after the topical application (less than 1% of the applied dose penetrates the stratum corneum). Thus, we suggest that the treatment design should be based on the combination of the antioxidants with an effective delivery system for topical application. 

Antioxidant liposomes, spherical nanoparticles consisting of phospholipid bilayer(s) with an aqueous compartment, possess the unique ability to effectively deliver both lipid soluble chemical antioxidants (in the lipid bilayer) and water-soluble chemical antioxidants (in the aqueous phase) [16, 17]. Moreover, liposomes rapidly penetrate the dermal barrier making the delivery of water-soluble compounds as effective as lipid-soluble drugs [18]. In contrast with other drug carriers such as DMSO [19], liposomes are absolutely harmless to human skin since they are composed of naturally occurring biodegradable phospholipids. In previous studies, it has been demonstrated that antioxidant liposomes with encapsulated NAC and antioxidant enzymes protect lung epithelial cells and reduce lymphocyte infiltration in rats intratracheally exposed to CEES [20–22]. We suggest that antioxidant liposomes could be useful as a multifunctional therapy since liposomal formulations similar to those used in the rat lung studies could also be protective in skin. In the present study, we tested the ability of several liposomal formulations containing water-soluble antioxidants (NAC or GSH) and/or lipid-soluble antioxidants (tocopherols or alpha-lipoic acid) to attenuate CEES toxicity after the dermal exposure. The protective effects of liposomes were initially studied in HaCaT human keratinocytes exposed to sulfur mustard analog, CEES. The most protective GSH-containing liposomes were then tested in a human epidermal model, EpiDerm.

## 2. Materials and Methods

### 2.1. Materials and Reagents

Stemline Keratinocyte Medium II (without L-glutamine, calcium-free), fluorimetric Caspase-3 Assay Kit, Sigma Luciferase ATP Determination kit, CEES, NAC, GSH, cholesterol, RRR-alpha-tocopherol, RRR-gamma-tocopherol, R-alpha-lipoic acid, DMSO, EDTA, and all organic solvents were obtained from Sigma Chemical Company (Sigma-Aldrich Inc., St. Louis, Mo). CellTiter 96 Aqueous One Solution Cell Proliferation Assay kit based on MTS (3-(4,5-dimethyl-2-yl)-5-(3-carboxymethoxyphenyl)-2-(4-sulfophenyl)-2H-tetrazolium, inner salt) was purchased from Promega Corporation (Madison, Wis). Soybean phospholipid mixture phospholipon 90G was obtained from American Lecithin Company (Oxford, Conn).

### 2.2. Cell Culture and Treatments

Spontaneously immortalized human keratinocytes HaCaT were purchased from Cell Lines Service (Eppelheim, Germany). HaCaT cells were cultured at 37°C in a humidified incubator with 5% CO_2_ in Stemline Keratinocyte Medium II (without L-glutamine, calcium-free) supplied with 10% fetal bovine serum Sigma Chemical Company (St. Louis, Mo). Adherent cells were subcultured over night in 96 well Costar tissue culture plates in the Keratinocyte Medium II and treated with CEES in the presence or absence of various concentrations of antioxidants or antioxidant liposomes as indicated in the figures. CEES was used only as fresh stock solutions in anhydrous DMSO, which was quickly mixed with culture medium immediately prior to incubation (final concentration of DMSO was 1% (vol./vol.)). CEES final concentration of 1 mM was chosen as it stably reduced HaCaT viability to 20–30% in our experimental settings (preliminary experiments). This observation is in accordance with findings published by other investigators [14, 15]. 

EpiDerm human skin model system EPI-200 and conditioning medium were purchased from MatTek Corporation (Ashland, Mass). EpiDerm is an *in vitro* human skin equivalent that consists of normal, human-derived epidermal keratinocytes (NHEK), which have been cultured to form a multilayered, highly differentiated model of the human epidermis (http://www.mattek.com/pages/products/epiderm). The EpiDerm tissues in a clear plastic 24-well plate were reconditioned overnight according to the manufacturer's instructions. CEES-containing media (freshly mixed) were applied topically (250 *μ*L per tissue) into the plate insert. Treatments (free antioxidant solutions or antioxidant liposomes) premixed in the culture media were applied by changing the bottom media. NAC and GSH are capable of scavenging mustard electrophiles in solution [23, 24]. In order to prevent direct interaction of the antioxidant liposomes with CEES, GSH- or NAC-containing liposomes were added into the bottom media, whereas CEES containing media were applied to EpiDerm tissues topically.

Water-soluble antioxidants (GSH or NAC) were prepared as 0.5 M stock solutions in PBS (pH adjusted to 7.4), filter-sterilized, and stored at 4°C for two weeks or less. Lipophilic antioxidants (alpha-tocopherol, gamma-tocopherol, or alpha-lipoic acid) were prepared as 50 mM stock solutions in anhydrous ethanol and stored at −20°C.

### 2.3. Antioxidant Liposome Preparation and Application

Antioxidant liposomes were prepared by extrusion of the phospholipid/cholesterol dry film emulsified in PBS [25]. Six types of antioxidant liposomes (see Table 1) were made by the extrusion technique using a small extruder apparatus purchased from Avanti Polar Lipids Inc. (Alabaster, Ala) according to manufacturer's instructions. Prior to extrusion, a lipid film was prepared in a 2 mL glass vial by mixing the lipid-soluble component stock solutions in chloromethane (phospholipon 90G, cholesterol with or without tocopherols or alpha-lipoic acid) with subsequent evaporation of the solvent under a nitrogen stream. The thin lipid film was then hydrated and detached by vigorous mixing in PBS (which did or did not contain water-soluble antioxidants GSH or NAC) at 30°C. Solubilized large multilamellar vesicles were then extruded through a polycarbonate membrane with 100 nm size pores (Avanti Polar Lipids Inc., Alabaster, Ala) at 50°C. The resulting liposomes, large unilamellar vesicles with a mean diameter 120 ± 30 nm, were used the next day or stored at 4°C for one week or less. Liposomes of this size provide relatively high antioxidants/phospholipids ratio and are not large enough to stimulate macrophages. Liposomes of this size have been successfully used to attenuate CEES toxicity in various models [20–22, 26]. Size distribution of the liposomes was monitored in PBS by using dynamic light scattering with a Nicomp 380 Particle Sizing Systems (Port Richey, Fla). If liposomes were stored for more than one day their size distribution, the main parameter of liposome stability, was monitored again prior to the treatment. All of our liposome preparations appeared stable for at least one week.

In the tissue culture experiments, antioxidant liposomes (Table 1) or antioxidant solutions/suspensions in PBS (as controls) were applied to HaCaT cells simultaneously with CEES. In the EpiDerm experiments, antioxidant liposomes (containing 200 mM NAC or GSH encapsulated) or antioxidant solutions in PBS (as controls) were applied to EpiDerm tissues via the bottom membrane (diluted 1 : 20 (v/v) in the bottom media, thus providing a “calculated” final concentration of 10 mM) and simultaneously with CEES, which was applied topically. Such application excluded direct contact between CEES and liposomes, therefore preventing possible absorption of CEES by liposomal phospholipids and subsequent hydrolysis of CEES by the aqueous liposomal interior. Thus, liposomes did not directly interfere with CEES toxicity. Overall design of these experiments was developed in accordance with the studies of CEES toxicity in the EpiDerm models [27–29] as well as with the studies of the protective effects of antioxidant liposomes in various models [20–22, 26].

### 2.4. Cell Viability Assays

The PI assay in the HaCaT cells was performed by a slight modification of the method described earlier [30]. Briefly, at the end of each experiment, cultured cells in 96 well dark plates with clear bottom (200 *μ*L of medium per well) were incubated with PI (final concentration 2 *μ*g/mL) at 37°C for 30 min. PI fluorescence in the dead cells was measured using a Fluostar Galaxy microplate reader (BMG, Germany) at an excitation wavelength of 485 nm and an emission wavelength of 650 nm. The results were calculated using the formula: viability (%) = 100 × (1– [(Test Sample – Low Control)/(High Control – Low Control)]), where Test Sample is fluorescence of the cells exposed to CEES or vehicle, Low Control is fluorescence of the cells in medium without CEES or vehicle (negative control, minimal fluorescence), High Control is fluorescence of the cells incubated in medium containing 1% Triton X-100 (positive control, maximal fluorescence).

The MTS assay in the HaCaT cells and in the EpiDerm tissues was performed using CellTiter 96 Aqueous One Solution (MTS solution) according to the manufacturer's instruction. Briefly, at the end of each experiment, cultured cells in clear plastic multiwell plates were incubated with CellTiter 96 Aqueous One Solution (diluted 1 : 20 (v/v) with medium) at 37°C for 1.5 hours. For HaCaT keratinocytes, the MTS solution was applied by complete change of the culture medium. For EpiDerm tissues, CellTiter 96 Aqueous One Solution was diluted into fresh bottom media and applied simultaneously with CEES or vehicle (applied topically). The formazan derived from MTS is water soluble (unlike MTT formazan product that needs to be dissolved in DMSO) and does not require additional washout or any separation of the cells prior to the OD measurement. The formazan product produced by viable cells was detected by a visual inspection, and the OD was measured at 590 nm with a Spectra Max Plus 384 microplate reader (Molecular Devices, Sunnyvale, Calif).

In our preliminary experiments, blank liposomes containing phospholipids and cholesterol only (see Table 1) did not show any effect on cell growth inhibition after 24 or 48 hour incubation; also, they did not attenuate the effect of CEES significantly (data not shown). Therefore, we used blank liposomes as a control.

### 2.5. Caspase-3 and ATP Assays

Caspase-3 assay (an apoptosis index) and intracellular ATP measurements (drastic ATP loss reflects necrosis) were performed by assaying cell lysates using the Caspase-3 Assay Kit and the Sigma Luciferase ATP Determination kit, respectively, (Sigma-Aldrich Inc., St. Louis, Mo) according to manufacturer's instructions. Caspase-3 and ATP were measured in the cell lysates in order to estimate relative contribution of apoptosis and necrosis in the cell death mechanism in the EpiDerm. Staurosporine (5 *μ*M) was used to induce apoptosis (positive control). To prepare cell lysates, EpiDerm tissues were carefully removed from insert membranes using a sterile forceps and placed in 1.5 mL microcentrifuge tubes. A volume of 100 *μ*L of Somatic cell ATP releasing reagent (Sigma-Aldrich Inc., St. Louis, Mo) was added to each tube. Each tissue was crushed into pieces using a small pestle. The homogenized mixture was vortexed briefly, and then frozen at −80°C. The next day the samples were allowed to thaw for 15 min at room temperature. Then the tubes were centrifuged at 4°C for 30 min at 16,000 ×g. The supernatant fluids, designated the tissue lysates, were transferred to fresh plastic tubes and were placed on ice until assayed. All assays were performed immediately upon preparation of tissue lysates.

### 2.6. Microscopic Examinations

At the end of incubation with CellTiter 96 Aqueous One Solution (containing MTS) EpiDerm tissues were rinsed with cold sterile PBS, removed from insert membranes using forceps, placed on aluminium foil and frozen at −80°C. For sectioning, each tissue was fixed between two thick layers of OCT (Optimal Cutting Temperature) compound. The “sandwich” was cut in half and fixated on a microtable with OCT compound in order to make vertical cuts. Tissue slices (25 *μ*m thickness) were made on Leica Rapid Sectioning Cryostat CM 1900 (Meyer Instruments Inc., Houston, Tex), put on glass slides and fixed with formalin solution (Sigma-Aldrich Inc., St. Louis, Mo) without additional staining. Slides were examined and photographed using a BX51 Olympus Microscope equipped with a CC12 digital camera (Olympus America Inc., Center Valley, Pa).

### 2.7. Fluorescent Microscopic Examinations

HaCaT cell density was adjusted to 2 × 10^5^/mL, and a 100 *μ*L aliquot of the cell suspension in media was placed in each well of an 8-well Lab-Tek chamber glass slide (Nunc, Rochester, NY). Vehicle (1% DMSO) or CEES (1 mM) in the presence or absence of 10 mM GSH (either encapsulated in the liposomes (GSH-liposomes) or nonencapsulated (free GSH)) were simultaneously added to chamber slides and incubated for 6 hour (to evaluate ROS generation due to CEES exposure) or 18 hours (to evaluate total thiol depletion due to CEES exposure) at 37°C in 5% CO_2_. The time period for the ROS monitoring experiment was relatively short in order to account for relatively short lifespan of ROS particles. At the end of the incubation (6 or 18 hours, resp.) 100x stock solution of 6-carboxy-2′,7′-dichlorodihydrofluorescein diacetate (carDCFH-DA; H_2_O_2_-sensitive probe for intracellular ROS monitoring) or 5-chloromethylfluorescein diacetate (CMF-DA; nonprotein thiol sensitive probe for intracellular thiols, including GSH) in DMSO was added (final carDCFH-DA or CMF-DA concentration was 10 *μ*M) and the slides were further incubated for 20 min at 37°C in the dark. 100 *μ*M tert-butyl hydroperoxide (TBHP) was used to induce oxidative stress in the absence of CEES or antioxidants (positive control). The cells were washed with cold PBS twice, observed, and digitally photographed using a MOTIC inverted phase contrast fluorescence microscope equipped with a Nikon Coolpix E4300 4-megapixel camera (Martin Microscope, Easley, SC). Vehicle-treated sample stained with carDCFH-DA were photographed as merged images (green fluorescence and phase contrast) in order to visualize live cells in the absence of fluorescence. The number of living cells (green fluorescence) was determined after counting ≥200 cells in the squares (4 squares per sample) of same size for each condition tested. Data were expressed as mean ± SD percent reduction from control.

### 2.8. Statistical Analyses

Data were analyzed by ANOVA followed with the Scheffe test for significance with *P* < .05 using SPSS 17.0 for Windows (Chicago, Ill). Results were expressed as the mean ± SD. In all the figures, mean values not sharing a common letter are significantly different (*P* < .05). Mean values sharing a common letter are not significantly different. The mean values and standard deviations of at least three independent experiments are provided in all the figures.

## 3. Results

### 3.1. The Protective Effect of Various Antioxidant Liposomes in HaCaT Cells

**Figure 1:**
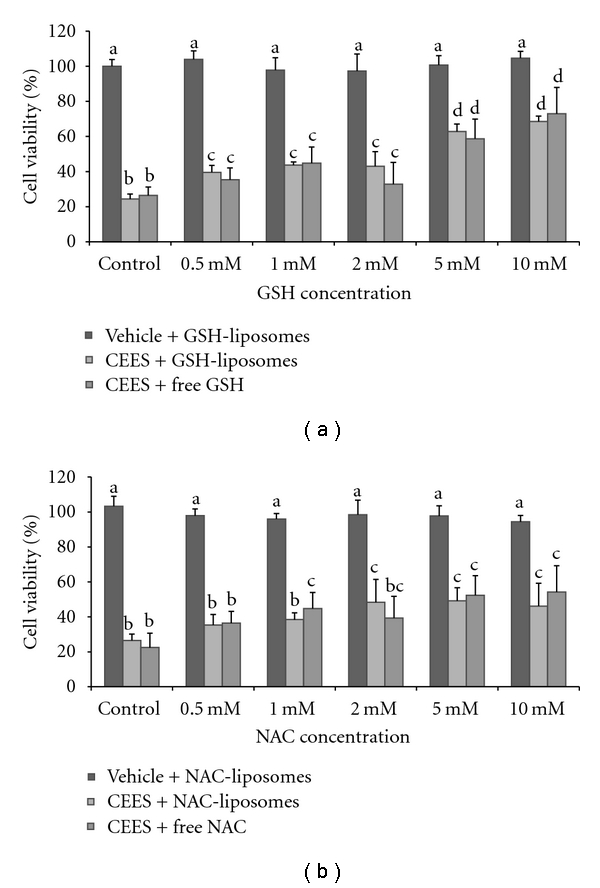
*Protective effects of “free” and liposome-encapsulated antioxidants in HaCaT cells*. GSH (a) or NAC (b), either “free” (as stock solutions in PBS) or encapsulated in the phospholipid-based liposomes (calculated final concentration as indicated), were applied to HaCaT cells simultaneously with 1 mM CEES or vehicle (1% DMSO); blank liposomes (no antioxidant encapsulated) or PBS were used as controls for GSH- or NAC-liposomes and for “free” GSH or NAC, respectively. Cells were incubated at 37°C for 18 hours. Cell viability was measured using the MTS assay (see Section 2). Mean values not sharing a common letter are significantly different (*P* < .05).

We first performed a series of preliminary experiments testing the effect of various antioxidant liposome formulations in HaCaT keratinocytes exposed to CEES. Six types of liposomes containing various antioxidants: Blank (no antioxidants), NAC, GSH, alpha-tocopherol (AT), gamma-tocopherol (GT), or alpha-lipoic acid (ALA) were used for the initial experiments in HaCaT cells.  Figure 1 shows HaCaT cell viability (MTS assay) after the exposure to 1 mM CEES and simultaneous treatment with “free” or liposome-encapsulated water-soluble antioxidants (NAC or GSH). The effects of antioxidant liposomes on the cell proliferation were measured by the MTS assay after 18 hour incubation (see Section 2). These results were confirmed with viability assays using the fluorescent probe propidium iodine (PI) that stains dead cells (data not shown). Both assays showed similar patterns for cell survival (PI) and for cell growth inhibition (MTS). Only water-soluble antioxidants (NAC and GSH) were effective in these experiments. Both 5 mM and 10 mM GSH-liposomes prevented CEES-induced decrease of cell viability in HaCaT keratinocytes (Figure 1(a)). In a similar experiment, NAC-liposomes also showed a dose-dependent increase in cell viability with the maximum at 10 mM, however, the effect was slightly lower compared to GSH-liposomes (Figure 1(b)). GSH-liposomes were the most protective since they increased cell viability from 28% (blank liposomes) to 67% (10 mM GSH, liposome encapsulated). However, the effects of liposome-encapsulated NAC and GSH were equal to the effects of nonencapsulated “free” NAC and GSH. The lipid-soluble antioxidants (alpha-tocopherol, gamma-tocopherol, or alpha-lipoic acid) were not protective when liposome-encapsulated or as suspensions in PBS (data not shown).

### 3.2. GSH Prevents ROS Generation and Thiol Depletion in HaCaT Cells

**Figure 2:**
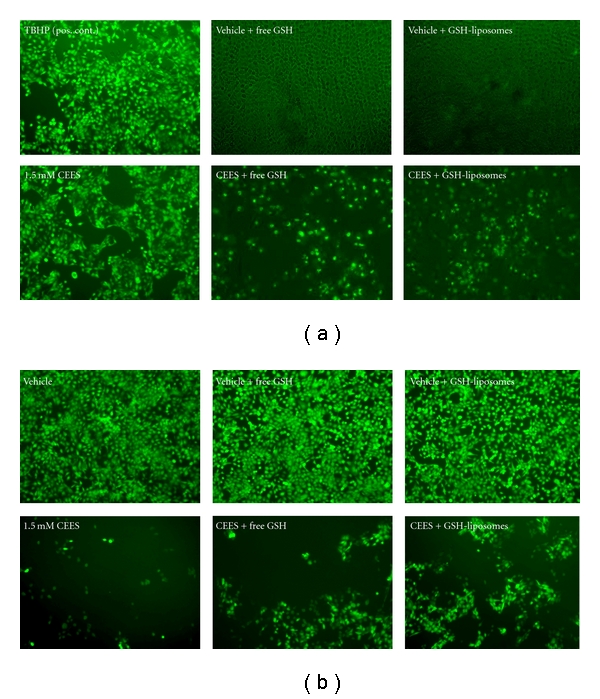
*GSH prevents ROS generation and thiol depletion in HaCaT cells*. HaCaT cells were treated with vehicle (1% DMSO) or 1.5 mM CEES (as indicated) in the presence or absence of “free” GSH or GSH-liposomes (as indicated), simultaneously. Cells were incubated at 37°C for 6 hours and then stained with 10 *μ*M carDCFH-DA to monitor ROS generation (a); alternatively, cells were incubated at 37°C for 18 hours and then stained with 10 *μ*M CMF-DA to access intracellular nonprotein thiols (b). 100 *μ*M TBHP (tert-butyl hydroperoxide) was used as a positive control (to induce oxidative stress). Live cells were photographed under a fluorescent microscope (200x magnification) equipped with a standard FITC filter. Vehicle-treated sample stained with carDCFH-DA (a) shows merged images (green fluorescence and phase contrast) in order to visualize live cells in the absence of fluorescence.

In order to show that CEES induces oxidative stress in the HaCaT cells we performed a series of fluorescent microscopy experiments monitoring ROS and thiol status of the CEES-treated cells in the presence and in the absence of GSH-liposomes (Figure 2). As evidenced by carDCFH-DA fluorescence, intracellular H_2_O_2_, a major marker of ROS generation, was enhanced upon the CEES treatment (6 hours); however, in the presence of GSH-liposomes or “free” GSH, ROS production was almost fully abolished (Figure 2(a)). CEES exposure (18 hours) also reduced average number of viable cells counted under microscope (14.7 ± 8.2% of control) and promoted total thiol depletion (Figure 2(b)); the effect was attenuated in the presence of GSH-liposomes (33.1 ± 9.5% of control), as well as in the presence of “free” GSH (28.4 ± 11.5% of control). These observations provide additional evidence that the GSH-liposomes effectively protect human keratinocytes exposed to CEES and attenuate CEES-induced ROS production and thiol depletion, although the effect of liposome-encapsulated GSH was similar to the effect of “free” GSH. These data also suggest that in HaCaT keratinocytes, GSH-liposomes provide effective intracellular GSH delivery. 

### 3.3. The Effects of Antioxidant Liposomes in the EpiDerm Model

EpiDerm tissue consists of organized basal, spinous, granular, and cornified layers of differentiating epidermal human keratinocytes that closely mimic human epidermis. At present, EpiDerm is one of the best available *in vitro* models of human skin and has successfully been used to study CEES toxicity *in vitro* [27, 28]. At first, we explored cytotoxic effect of CEES in EpiDerm tissues. A dose-dependent decrease of cell viability due to CEES (0.5–5.0 mM) exposure was observed (data not shown). CEES concentration of 2.5 mM reduced cell viability within the EpiDerm tissues to 48 ± 8.6%, and this concentration was chosen for the following experiments to study the effect of antioxidants.

**Figure 3:**
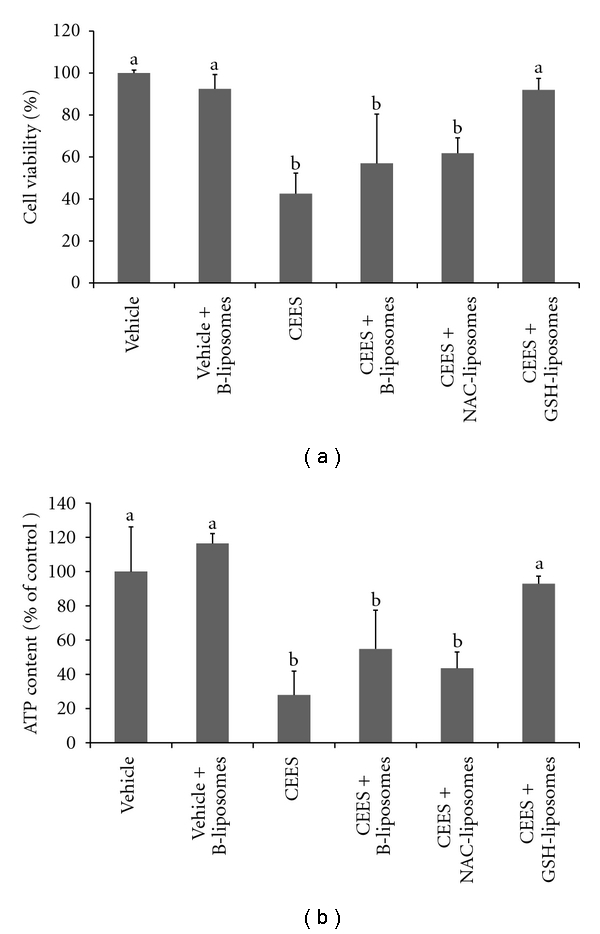
*Protective effects of antioxidant liposomes in the EpiDerm model*. EpiDerm tissues were exposed topically to 2.5 mM CEES or vehicle (1% DMSO) in the absence or presence of Blank liposomes (B-liposomes), NAC-liposomes, and GSH-liposomes (as indicated) applied simultaneously with CEES or vehicle, but into the bottom media; the tissues were incubated at 37°C for 18 hours. Final “calculated” concentration of GSH or NAC was 10 mM (see Section 2). Cell viability was monitored by the MTS assay (a). Cellular ATP was assayed in the tissue homogenates by the ATP assay (b). Means not sharing a common letter are significantly different (*P* < .05).

We tested the protective effect of GSH- and NAC-containing liposomes in EpiDerm tissues exposed to CEES. We found that only GSH-liposomes increased cell viability (Figure 3(a)) and reduced ATP depletion (Figure 3(b)) in the EpiDerm tissues topically exposed to 2.5 mM CEES. GSH-liposomes added immediately after CEES provided an extremely effective countermeasure to CEES toxicity since both cell viability (Figure 3(a)) and ATP level (Figure 3(b)) did not significantly decrease from their original levels as observed in cells treated with vehicle alone. The attenuation of ATP depletion in keratinocytes evidenced that GSH-liposomes reduce necrosis induced by CEES.

### 3.4. The Effect of Antioxidant Liposomes on Apoptosis in the EpiDerm Model

**Figure 4:**
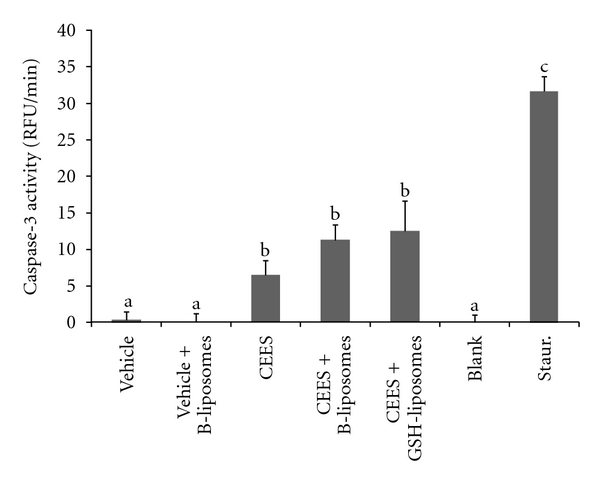
*GSH-liposomes do not reduce apoptosis in the EpiDerm model*. EpiDerm tissues were exposed topically to 2.5 mM CEES or vehicle (1% DMSO) in the absence or presence of Blank liposomes (B-liposomes) or GSH-liposomes (as indicated) applied simultaneously with CEES or vehicle, but into the bottom media; the tissues were incubated at 37°C for 18 hours. Final “calculated” concentration of GSH was 10 mM (see Section 2). Caspase-3 activity was assayed in the tissue homogenates by the caspase-3 assay (see Section 2). Blank: blank buffer (negative control); Staur.: 5 *μ*M staurosporine (positive control). Means not sharing a common letter are significantly different (*P* < .05).

We also evaluated the ability of GSH-liposomes to influence apoptosis in CEES-treated EpiDerm tissues using the caspase-3 assay (Figure 4). CEES and HD induce both apoptosis and necrosis in human cell lines including HaCaT keratinocytes. However, relatively low doses of HD induce mostly apoptosis, whereas higher doses promote necrosis [31, 32]. Thus, it is likely that CEES ≥2.5 mM would promote mostly necrosis with only limited induction of apoptosis. Indeed, at a level of 2.5 mM, CEES induced a relatively low level of apoptosis in EpiDerm (Figure 4). Considering the marked loss of ATP (Figure 3(b)), necrosis, but not apoptosis is likely the major cause of cell death at this level of CEES. Figure 4 also shows that GSH-liposomes did not inhibit the apoptosis induced by 2.5 mM CEES. These experiments suggest that GSH-liposomes are effective in blocking CEES toxicity and necrosis, but do not prevent apoptosis in the EpiDerm model.

### 3.5. The Protective Effect of GSH-Liposomes in EpiDerm Model (Posttreatment)

**Figure 5:**
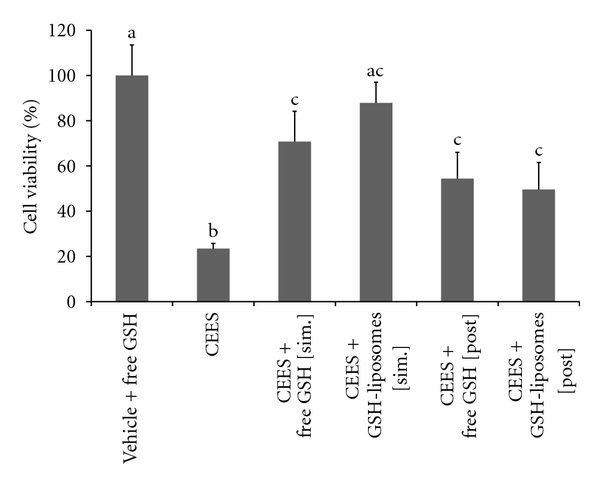
*Protective effects of GSH-liposomes or free GSH in EpiDerm (posttreatment)*. EpiDerm tissues were exposed topically to 2.5 mM CEES or vehicle (1% DMSO) in the absence or presence of Blank liposomes (B-liposomes) or GSH-liposomes (as indicated) applied simultaneously with CEES or vehicle, but into the bottom media (sim: simultaneous application; post: 1 hour post-treatment). Final “calculated” concentration of GSH was 10 mM (see Section 2). Cell viability was monitored after 18 hours by the MTS assay (see Section 2). Means not sharing a common letter are significantly different (*P* < .05).

In order to test the potential protective effect of GSH-liposomes under posttreatment conditions, we performed an experiment, in which NAC- or GSH-liposomes were applied to EpiDerm one or two hours after the CEES (2.5 mM) exposure. Only GSH-liposomes and “free” GSH showed significant protection in this experiment (Figure 5). In the EpiDerm tissues, CEES exposure reduced cell viability to 24 ± 5%; GSH-liposomes applied simultaneously with CEES altered cell viability to 87 ± 9%; GSH-liposomes applied one hour after CEES altered cell viability to 52 ± 11%; GSH-liposomes applied two hours after CEES altered cell viability to 32 ± 12%. Although the latter (two hours) effect was not significant, the effect of one-hour posttreatment with GSH-liposomes was statistically significant, and the toxicity of CEES was attenuated substantially (two-fold difference). NAC-liposomes did not show any statistically significant protective effect in the posttreatment experiments (data not shown).

### 3.6. The Influence of GSH-Liposomes on Cellular Morphology Changes in EpiDerm

**Figure 6:**
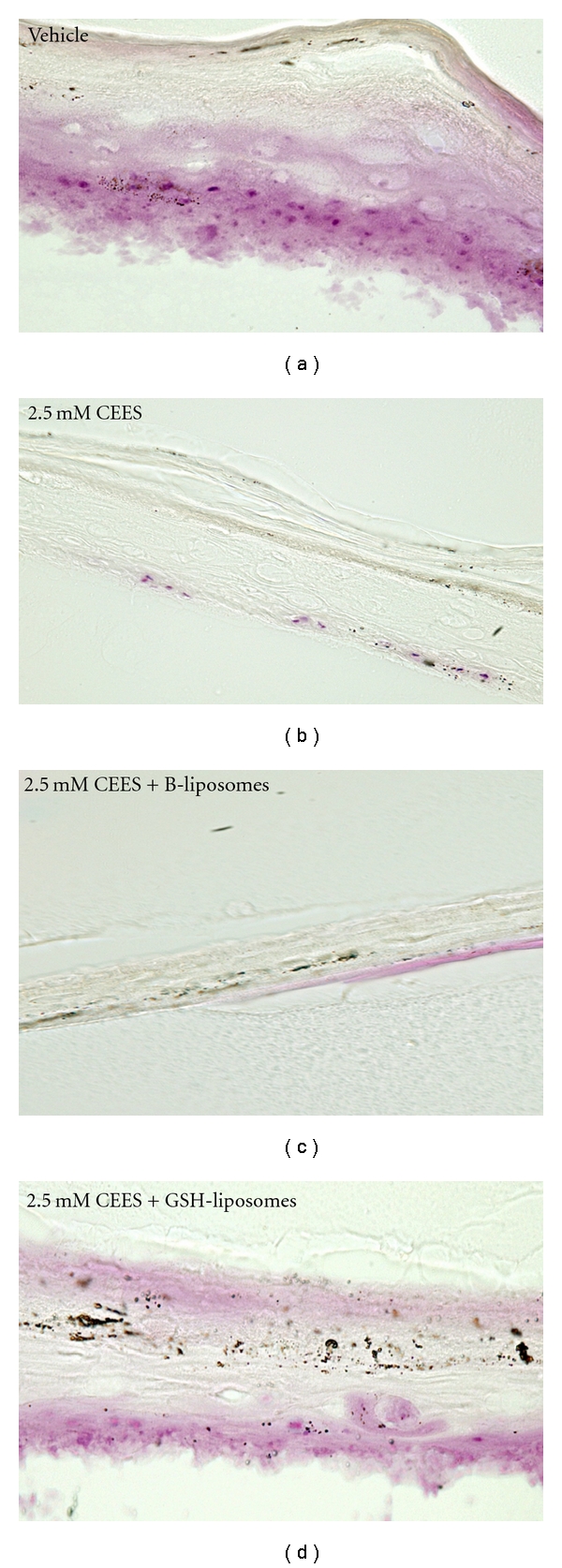
*Protective effect of GSH-liposomes in the EpiDerm model*. EpiDerm tissues were topically exposed to vehicle (1% DMSO) or 2.5 mM CEES in the absence or presence of GSH-liposomes or Blank liposomes (B-liposomes). After the MTS assay EpiDerm tissues were frozen, dissected, and photographed under a light microscope with 400x magnification (see Section 2). Violet color reflects the accumulation of the MTS formazan product in the proliferating keratinocytes. Note the disruption of the keratin filament structure in the stratum corneum in the presence of CEES, and the increase of proliferating (violet) basal keratinocytes in the presence of GSH-liposomes.

It is known that HD and its analogs induce morphological changes in keratinocytes, in particular, HD affects size and shape of the cells and induces fragmentation of the extracellular matrix [3]. In order to determine whether GSH-liposomes influence keratinocyte morphology, we performed microscopic examinations of EpiDerm tissues incubated with CEES in the presence or absence of GSH-liposomes; tissues were stained with MTS at the end of the incubation to visualize viable cells. After CEES (2.5 mM) exposure the apical side of the keratinocyte multilayer showed the matrix disassembly (the cells lost their compact structure within the tissue and prominent gaps appear in the matrix); the integrity of the cell multilayer was markedly disturbed and the majority of proliferating cells were detached and lost. The remaining cells formed a dense structure with a very limited number of live cells. The remaining live cells appeared violet in color due to the reduction of MTS dye by functioning mitochondria; these cells showed size reduction and round shape (Figure 6). This observation is in agreement with recent study of CEES toxicity in the EpiDerm full-thickness skin equivalents [29]. Treatment with blank liposomes (composed with phospholipids only) did not affect the CEES-exposed cells, however, marked changes were observed upon simultaneous treatment with CEES and GSH liposomes (Figure 6). The number of live keratinocytes increased as observed by the MTS staining; some live cells retained normal shape; however, the overall integrity of the tissue was somewhat lower if compared to the control (tissues exposed to vehicle only).

## 4. Discussion

Our studies with the human epidermal model, EpiDerm, showed that GSH, both “free” and “liposome encapsulated,” attenuated toxicity induced by CEES, a mustard gas analog. In our experimental settings, antioxidant liposomes and CEES were applied separately (topically and to the bottom media, resp.) in order to prevent their direct interaction and scavenging of CEES by the liposomes. Systemic application of free GSH would require consumption of large amounts of solid or liquid antioxidants because hydrophilic compounds poorly penetrate the stratum corneum; therefore, antioxidant liposomes would provide a reasonable alternative, especially after the dermal exposure. Moreover, the liposome design presents an additional advantage as it allows encapsulating of multiple lipophilic and hydrophilic components. For instance, antioxidants can be combined with anti-inflammatory drugs as inflammation plays a role in the development of skin lesions [33].

At a relatively high CEES dose (2.5 mM), the major cause of cell death in our studies was necrosis with only a slight additional effect caused by apoptosis. Our results indicated that the GSH liposomes not only enhanced cell viability in EpiDerm tissues exposed to CEES, but also reduced necrosis as evidenced by inhibition of ATP depletion induced by CEES exposure. On the other hand, as shown by the caspase-3 activity measurements, GSH liposomes had no significant effect on CEES-induced apoptosis (2.5 mM CEES). This is in accord with numerous studies of CEES or HD toxicity to various cell lines including human keratinocytes [3]. In endothelial cells and lymphocytes [6, 34, 35] and also in HaCaT keratinocytes [31, 32], relatively low doses of HD induced mostly apoptosis, whereas higher doses promoted necrosis. Chiarugi, [36] has shown that a potent alkylating agent, if applied to the cells at a sufficient level, induces a “programmable” form of necrosis due to a genotoxic stress. Necrosis is a more inflammatory form of cell death. In epidermal keratinocytes, necrosis, but not apoptosis, promotes inflammatory responses [37–40]. In various skin models, the open wounds due to HD exposure were highly susceptible to infections and showed many typical characteristics of necrotic tissues [2, 31]. Thus, in the search for therapeutic countermeasures to HD, it is important to show that the prospective treatment not only increases cell viability but also reduces necrosis. In our recent work, we have discussed an “apoptosis-to-necrosis switch” in the HaCaT cells exposed to CEES [41]. 

The molecular mechanisms of CEES/HD toxicity in human skin have not been fully investigated. Recent findings strongly suggest, however, that these mechanisms are complex; they involve DNA and other macromolecular damage, oxidative stress and inflammation [3, 33]. It is widely accepted that ROS generation and oxidative damage play a critical role in these mechanisms. In the EpiDerm model, GSH liposomes showed significant protective effect even when applied 1 hour after CEES exposure. This observation further confirmed previous *in vitro* studies of CEES/HD toxicity, which have demonstrated that the antioxidants that reduce oxidative stress and prevent thiol depletion also attenuate CEES toxicity, whereas oxidants (hydrogen peroxide) [4] and agents that deplete intracellular glutathione (buthionine sulphoximine) [6, 42] sensitize human cell lines, in particular HaCaT [42], to CEES/HD toxicity. Thus, our study provides additional evidence of the critical role of oxidative stress in the pathogenesis of CEES/HD-induced injury in the skin. Although the protective effects of various antioxidants are well documented both *in vitro* and *in vivo* [2, 3], it is possible that in the human skin, the antioxidants are effective only when applied prior to or short time after (about 1 hour) CEES/HD exposure. For instance, in human endothelial cells, NAC altered cell viability only when applied simultaneously or prior to HD [6]. Similarly, in a guinea pig model, superoxide dismutase, a powerful antioxidant enzyme, reduced skin lesions only when applied prior to HD exposure [43]. It should be taken into account that the main direct chemical impact of the alkylating agents, such as HD or CEES, within the cell is attributable to the DNA damage derived from rapid alkylation of guanines by mustard sulfonium ions [3, 24]; NAC and GSH (as nucleophilic scavengers) are capable of scavenging mustard electrophiles in the cytosol [23, 24]. However, this direct detoxification is possible only during a short time after the exposure, and other nucleophilic scavengers, for example, thiopurines [23] or exogenous peroxidases [44], are more effective in degrading of HD or CEES. Nevertheless, antioxidants and antioxidant liposomes have shown a great potential in preventive treatment of CEES/HD toxicity [3–5, 9, 10, 20, 21, 26, 34, 42, 45–48]. In addition, it is well known that mammalian skin cells exposed to CEES/HD release proinflammatory cytokines [27, 28, 37, 49] and other immune stimulators [50]. These immune factors mediate inflammatory responses that contribute to the development of skin lesions [33]. Therefore, in a long run, it is critical not only to protect keratinocytes from immediate cell injuries, but also to reduce the inflammatory responses in the skin [3, 33]. This approach will require deeper understanding of the molecular mechanisms of CEES/HD toxicity in multicell-type and animal models. 

In summary, we found that GSH liposomes have shown a protective effect (viability increase, ATP content protection, oxidative stress reduction) in the EpiDerm model exposed to CEES, a close analog of mustard gas. The molecular mechanisms of the protective effect of GSH-liposomes involved reduction of necrosis (but not apoptosis). In addition, GSH liposomes attenuated ROS generation and intracellular thiol depletion that were induced by CEES. We suggest that in the EpiDerm model, GSH-liposomes protect the cells from necrosis not only by scavenging of CEES electrophiles, but also by reduction of oxidative stress and inflammation. The hypothesis, however, needs more evidence, and that is being addressed in a separate study. Although the protective effect of liposome-encapsulated GSH was limited in time and not statistically different to the effect of “free” GSH, we suggest that preventive or “quick-response” antioxidant therapy may be a useful strategy against mustard toxicity in human skin, especially considering the fact that water-soluble antioxidants cannot penetrate epidermis if applied to the skin as a solution, whereas the liposomes possess superior delivery abilities in dermal applications [18]. Taking into account previously described antioxidant liposome-derived protection in lung tissues [20–22, 26], we suggest that a multifunctional liposomal formulation containing encapsulated antioxidants will be effective against sulfur mustard toxicity both in the skin and in the lung.

## Figures and Tables

**Table 1:** Liposome composition.

Type of liposomes						
	Blank	GSH	NAC	AT	GT	ALA
Lipid components	Concentration (mole fractions)					

Phospholipon 90G	0.71	0.71	0.71	0.70	0.70	0.70
cholesterol	0.29	0.29	0.29	0.28	0.28	0.28
*α*-tocopherol	0	0	0	0.02	0	0
*γ*-tocopherol	0	0	0	0	0.02	0
*α*-lipoic acid	0	0	0	0	0	0.02

Aqueous* components	Concentration (mM)					

GSH	0	200	0	0	0	0
NAC	0	0	200	0	0	0

*All aqueous components were used as solutions in sterile PBS; liposomes that do not contain aqueous components were prepared with sterile PBS also.

Blank: Blank liposomes (contain no antioxidant); GSH: reduced glutathione; NAC: N-acetyl-L-cysteine; AT: alpha-tocopherol; GT: gamma-tocopherol; ALA: alpha-lipoic acid.

## Abbreviations

ALA: Alpha-lipoic acid
ATP: Adenosine-5′-triphosphate
AT: Alpha-tocopherol
carDCFH-DA: 6-carboxy-2′,7′dichlorodihydrofluoresceindiacetate
CEES: 2-chloroethyl ethyl sulphide
CMF-DA: 5-chloromethylfluorescein diacetate
GSH: Reduced glutathione
GSSG: Oxidized glutathione
GT: Gamma-tocopherol
HD: Sulphur mustard
MTT: 3-(4,5-dimethylthiazol-2yl)-2,5-diphenyltetrazolium bromide
MTS: 3-(4,5-dimethylthiazol-2-yl)-5-(3-carboxymethoxyphenyl)-2-(4-sulfophenyl)-2H-tetrazolium, inner salt
NAC: N-acetyl-L-cysteine
TBHP: Tert-butyl hydroperoxide
TNF-alpha: Tumor necrosis factor-alpha.
